# The extreme hyper-reactivity of Cys94 in lysozyme avoids its amorphous aggregation

**DOI:** 10.1038/s41598-018-34439-y

**Published:** 2018-10-30

**Authors:** Alessio Bocedi, Giada Cattani, Claudia Martelli, Flora Cozzolino, Massimo Castagnola, Pietro Pucci, Giorgio Ricci

**Affiliations:** 1 Department of Chemical Sciences and Technologies, University of Rome “Tor Vergata”, Rome, Italy; 2 Istituto di Biochimica e Biochimica Clinica, Università Cattolica and Istituto di Chimica del Riconoscimento Molecolare, Consiglio Nazionale delle Ricerche, Rome, Italy; 3 CEINGE Biotecnologie Avanzate and Department of Chemical Science, University of Naples “Federico II”, Naples, Italy

## Abstract

Many proteins provided with disulfide bridges in the native state undergo amorphous irreversible aggregation when these bonds are not formed. Here we show that egg lysozyme displays a clever strategy to prevent this deleterious aggregation during the nascent phase when disulfides are still absent. In fact, when the reduced protein assembles into a molten globule state, its cysteines acquire strong hyper-reactivity towards natural disulfides. The most reactive residue, Cys94, reacts with oxidized glutathione (GSSG) 3000 times faster than an unperturbed protein cysteine. A low p*K*_a_ of its sulfhydryl group (6.6/7.1) and a productive complex with GSSG (*K*_D_ = 0.3 mM), causes a fast glutathionylation of this residue (t_1/2_ = 3 s) and a complete inhibition of the protein aggregation. Other six cysteines display 70 times higher reactivity toward GSSG. The discovery of extreme hyper-reactivity in cysteines only devoted to structural roles opens new research fields for Alzheimer’s and Parkinson diseases.

## Introduction

The aggregation of misfolded proteins, often generating amyloid fibrils or amorphous aggregates, is a phenomenon associated with over 20 diseases including Parkinson’s and Alzheimer diseases and type 2 diabetes mellitus^1–5^. Interestingly, disulfide bonds are present in more than 50% of proteins involved in amyloidosis^6^ and their reduction are often the origin of this phenomenon^7–9^. Lysozyme (Lyz), one of the most studied protein about its folding mechanism^10^, is an enzyme which *in vitro* forms similar aggregates when its four disulfides are broken by reduction^7^, so it is not clear why aggregation does not occur *in vivo* during the nascent phase when these disulfides are still to be formed. Motivated by the above argument, we explore here the possibility that some unknown expedient have been developed during evolution to avoid the protein aggregation and favor the correct folding. It is well known that the oxidative folding of lysozyme as well as of other proteins occurs in the endoplasmic reticulum promoted by an unusual high concentration of GSSG (about 0.4 mM) and the possible assistance of the protein disulfide isomerase (PDI) involved in the rearrangement of incorrect disulfides^11,12^. This process, without PDI and only in the presence of glutathione/oxidized glutathione (GSH/GSSG) (in a ratio similar to that found in the endoplasmic reticulum) is really fast and a number of pioneering studies reported that the early first disulfide appears in less than one min and three disulfides within a few minutes^13–16^. All these investigations were mainly finalized to characterize the temporal sequence of the disulfide formation and their identification but, in our opinion, some very important details have not been adequately enlightened. What is the true incipit of the oxidative folding, i.e. the identity of the cysteine that first interacts with GSSG? Is this residue characterized by a normal or unusual reactivity toward GSSG compared to a free cysteine? Does the early glutathionylation of this residue influence the aggregation process? Does exist an increased kinetic propensity of the other protein cysteines to form the native disulfides exhibiting a particular hyper-reactivity towards GSSG? As a matter of fact, structural cysteines only devoted to form disulfides have been always considered as “neutral” actors in the oxidative folding of many proteins. We demonstrate here that some of these residues may play a crucial and active role in the nascent phase of lysozyme to prevent deleterious protein aggregation exhibiting extraordinary reactivity toward GSSG. The exceptionality of this phenomenon is that this hyper-reactivity appears when the reduced lysozyme acquires the molten globule state i.e. when only about 30% of secondary and tertiary structures is formed, revealing, for the first time, that this rudimental build may accomplish sophisticated functions. A similar scenario has been recently found in the molten globule state of reduced albumin where Cys75 becomes more than 300 times more reactive towards GSSG^17^.

## Results and Discussion

### Interaction of GSSG with the reduced lysozyme

**Figure 1:**
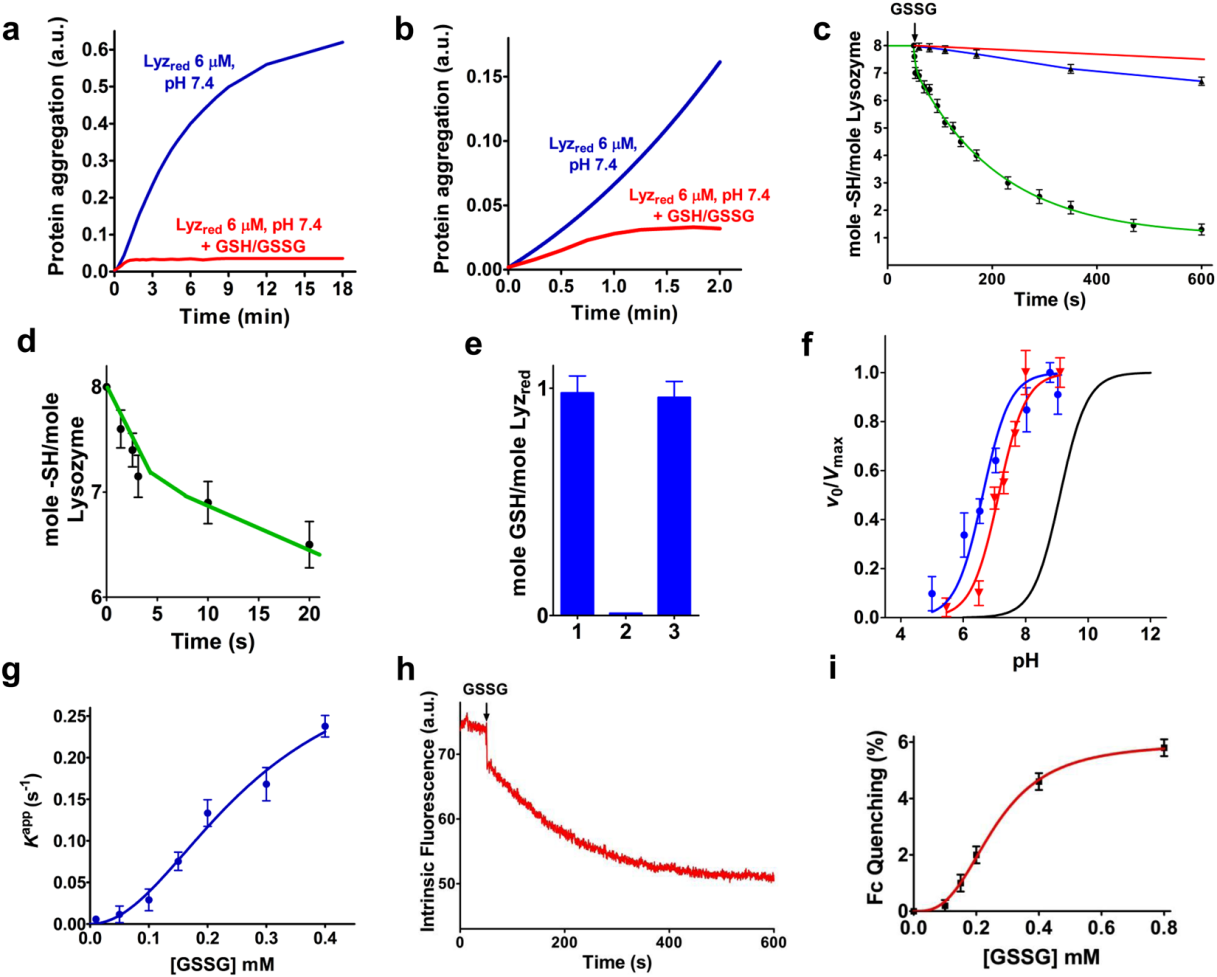
The effect of GSH/GSSG on Lyz_red_ aggregation, p*K*_a_ of Lyz_red_ cysteines and evidence for a transient Lyz_red_-GSSG complex. (**a**) *Blue line*: Lyz_red_ (6 µM) incubated in 0.2 M urea (37 °C). *Red line*: Same Lyz_red_ solution after immediate addition of GSH/GSSG (2 mM/0.4 mM). (**b**) Expanded kinetics of the experiment shown in (**a**). (**c**) *Green line*: Disappearance of Lyz_red_ sulfhydryl groups after addition of 0.4 mM GSSG to a 1.25 µM Lyz_red_ solution (10 µM –SH groups) at pH 7.4 and 0.2 M urea (25 °C). *Blue line*: Disappearance of the sulfhydryl groups of free cysteine (10 µM) incubated with 0.4 mM GSSG as in the experiment with Lyz_red_ (pH 7.4, 25 °C). *Red line*: Theoretical sulfhydryl groups disappearance of an unperturbed protein cysteine (10 µM) during the reaction with 0.4 mM GSSG. (**d**) Expanded kinetics of the experiment shown in **c**. (**e**) Reduced GSH produced after 10 s incubation of Lyz_red_ with 0.4 mM GSSG at pH 7.4. Titration of GSH using NBD-Cl and glutathione transferase P1-1 (GSTP1-1) (see Methods) (*column 1*). Same experiment in the absence of GSTP1-1 (*column 2*). Same experiment in which the produced GSH was titrated with DTNB after filtration of the mixture on Amicon Ultra 10 kDa cut-off filter to remove the protein (*column 3*). (**f**) Average p*K*_a_ of the seven reactive cysteines in Lyz_red_ as calculated using DTNB (p*K*_a_ = 6.6) (*blue line*); average p*K*_a_ of the four reactive cysteines using NBD-Cl (p*K*_a_ = 7.1) (*red line*). *Black line* is the theoretical curve for unperturbed cysteine (p*K*_a_ = 9.1). *v*_0_/*V*_max_ are the initial velocities normalized to those at full deprotonation (see Methods). (**g**) Apparent first order kinetic constants for the reaction of the most hyper-reactive cysteine with variable GSSG concentrations at pH 7.4 and 25 °C. (**h**) Quenching of the intrinsic fluorescence at 340 nm (λ_ex_ = 295 nm) of Lyz_red_ (1.25 µM, pH 7.4) after addition of GSSG 0.4 mM (25 °C). See (c) for comparison. The very fast fluorescence perturbation after the addition of GSSG occurs within one second. (**i**) The dependence of the fast fluorescence perturbation (occurring within 1 s) on the GSSG concentration. The error bars represent the S.D. from three independent experiments.

**Figure 2:**
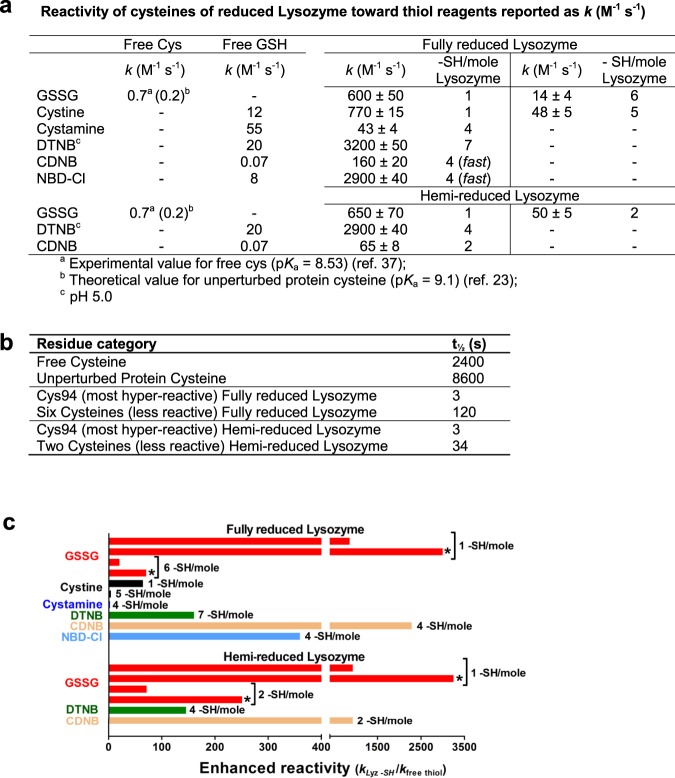
Reactivity of Lyz_red_ toward different disulfides and thiol reagents. (**a**) Second order kinetic constants *k* (M^−1^ s^−1^) for the reaction of the cysteines of Lyz_red_ and hemi-reduced Lyz, free Cys and free GSH with natural disulfides and other thiol reagents calculated at pH 7.4 and 25 °C (DTNB at pH 5.0). Errors are reported as S.D. from five independent experiments. (**b**) t_1/2_ for the reaction of cysteines of Lyz_red_ and hemi-reduced Lyz with GSSG at pH 7.4 and 25 °C. (**c**) Second order kinetic constants of Lyz_red_ and hemi-reduced Lyz toward GSSG normalized to the corresponding constants found for free Cys (0.7 M^−1^ s^−1^) or ($\ast$) calculated for unperturbed protein Cys (0.2 M^−1^ s^−1^) (see Methods section). All other bars represent the second order kinetic constants of Lyz_red_ and hemi-reduced Lyz in its reactions with other disulfides or thiol reagents normalized to the corresponding constants calculated for GSH. Note that electrostatic factors may have a critical role in determining the different kinetic constants for the reaction of free GSH (negatively charged) with cystine (neutral) and cystamine (positively charged).

While the native oxidized lysozyme (Lyz_ox_) is a protein which displays high solubility and good stability in solution over time, its reduced form (Lyz_red_) is highly unstable and when incubated at pH 7.4, a relevant aggregation occurs within a few minutes (Fig. 1a). Although the role of –SH groups in this phenomenon has previously been investigated^7,18–20^, the effect due to the addition of GSH and GSSG, at the concentrations similar to those found in the endoplasmic reticulum (2 mM and 0.4 mM, respectively)^21^ was not deeply examined in the past. Interestingly, a relevant decrease (about 60%) of the rate of aggregation occurs within a few seconds from the addition of the GSH/GSSG solution and a complete inhibition is achieved within one min (Fig. 1a,b). This evidence is quite surprising; a free cysteine would require a much longer time to react quantitatively with GSSG. Indeed, the second order kinetic constant for this reaction is 0.7 M^−1^ s^−1^ (pH 7.4 at 25 °C)^17,22^ (Fig. 2a) and assuming that at least 50% of one or a few protein cysteines must to be oxidized to avoid aggregation, an evident effect could not be observed before 40 min. An unperturbed protein cysteine would require even much longer time (about 140 min) as its p*K*_a_ is 9.1^23^, so the estimated *k* is about 0.2 M^−1^ s^−1^ (Fig. 2a). So the very fast inhibition of the lysozyme aggregation shown in Fig. 1 (panels a and b) suggests that at least one or more cysteines in lysozyme could have an unknown and particular hyper-reactivity toward GSSG. A fast interaction of a few cysteines of Lyz_red_ with GSSG has been previously reported by several authors^13,16^. However, an in depth kinetic analysis, a comparison of the observed kinetic constants with that of a free cysteine with GSSG, and a correlation with the inhibition of the protein aggregation have not been made. Thus, we first studied the kinetics and stoichiometry of this reaction by following the disappearance of the protein sulfhydryl groups after the addition of GSSG. A very fast reaction (within 8–10 seconds) involving only one protein cysteine was initially observed. The reaction was then followed by a second slower near monophasic trend involving six remaining cysteines (Fig. 1c,d). The first event can be unequivocally identified as a rapid glutathionylation of a single cysteine. In fact, after only ten seconds of reaction between Lyz_red_ and 0.4 mM GSSG, one equivalent of reduced GSH per mole of enzyme was released in solution (Fig. 1e).

The second order kinetic constant for the first oxidative event (600 ± 50 M^−1^ s^−1^) is about 850 fold higher than that of a free cysteine toward GSSG (0.7 M^−1^ s^−1^). The average second kinetic constant for the subsequent reaction of six remaining cysteines (14 ± 4 M^−1^ s^−1^) (Fig. 2a) is also more than one order of magnitude higher than that of a free cysteine (Fig. 2b). If the comparison is made considering the theoretical reactivity of an unperturbed protein cysteine toward GSSG (p*K*_a_ = 9.1, *k* = 0.2 M^−1^ s^−1^)^23^, these incremental factors becomes 3000 and 70, respectively (Fig. 2c).

### Lowered p*K*_a_ and reversible complex Lyz_red_-GSSG cause hyper-reactivity

**Figure 3:**
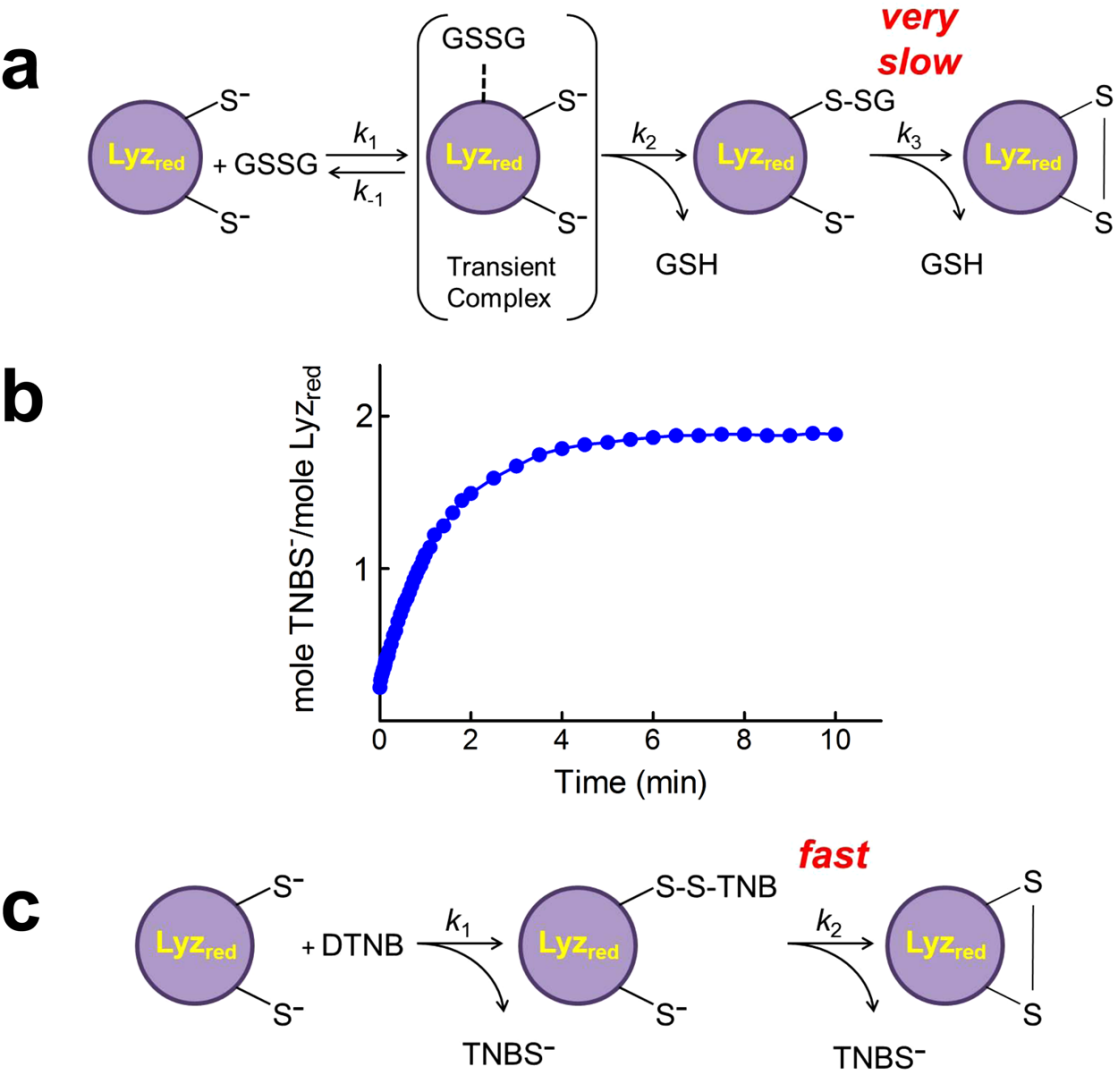
Reaction of Lyz_red_ with stoichiometric amounts of GSSG or DTNB. (**a**) Representative reaction scheme of Lyz_red_ with stoichiometric GSSG. Note that only the deprotonated sulphydryl groups are the reactive species. (**b**) Time course of release of TNBS^−^ when 1.25 µM DTNB reacts with 1.25 µM Lyz_red_ at pH 5.0. (**c**) Representative reaction scheme of Lyz_red_ with stoichiometric DTNB.

What is the cause of this hyper-reactivity? Given that only the deprotonated form of a thiol group is active in the thiol-disulfide exchange reactions (Fig. 3a), one possibility is that the p*K*_a_ of these protein cysteines is lower than that of a free cysteine.

The measured average p*K*_a_ value of the seven reactive cysteines with 5,5′-dithiobis(2-nitrobenzoic acid) (DTNB) (Fig. 1f) is 6.6, about 2.5 units lower than that of a “unperturbed” protein cysteine (9.1), while it results 7.1 for the four reactive cysteines with 4-chloro-7-nitrobenzofurazane (NBD-Cl) as thiol reagent (Fig. 1f). Both these values confirm a widespread increased acidity for most of the reduced cysteines. We note, however, that even assuming the lowest average p*K*_a_, i.e. 6.6, this would only cause, at pH 7.4, about 40 fold increased reactivity toward GSSG, a value approaching that observed experimentally for 6 cysteines in Lyz_red_ but far from the thousands times enhanced reactivity found in a single residue (more details for this conclusion are reported in the Supplemental Discussion). We thus argue that an additional kinetic improvement must be present to trigger the astonishing enhanced reactivity toward GSSG found in one single cysteine. One possibility is the occurrence of a productive reversible complex Lyz_red_-GSSG which would speed the glutathionylation in a way resembling what occurs in the active site of an enzyme when two substrates interact productively. A convincing indication for this hypothesis was given by evaluating the rates of disappearance of the single hyper-reactive cysteine at different GSSG concentrations (Fig. 1g). In fact, the saturation behavior suggests a cooperative reversible binding of GSSG with S_0.5_ of 2.7 × 10^−4^ M. A very similar trend was observed by using a fluorescence approach. As shown in Fig. 1h the addition of 0.4 mM of GSSG to Lyz_red_ causes a very fast intrinsic fluorescence perturbation at 340 nm, which occurs within 1 second (an event faster compared to the glutathionylation event), compatible with the formation of a reversible Lyz_red_-GSSG complex. The dependence of this very fast fluorescence perturbation on the different GSSG concentrations confirms a saturation trend and also a cooperative behavior with S_0.5_ of 3.1 × 10^−4^ M (Fig. 1i). This first event is followed by a second slower and more prominent fluorescence perturbation which is likely due to multiple glutathionylation events and intramolecular disulfide bond formation (Fig. 3a). In fact, quenching of fluorescence parallels the oxidation of the protein cysteines (see Fig. 1c,h).

### Mass spectrometry analysis identifies the hyper-reactive cysteine

Mass spectrometry analysis confirmed the existence of the surprising hyper-reactive residue and allowed its identification. Lyz_red_ was reacted with 0.4 mM GSSG at pH 7.4 for only ten seconds. Then 0.25 mM bromopyruvic acid was added to alkylate within 1–2 seconds the residual protein cysteines. A few drops of formic acid were added to lower the pH to 2.5. After a desalting step, the enzyme was subjected to limited proteolysis with pepsin followed by LC-MS/MS analysis. This procedure allowed to identify the glutathionylated residue as Cys94 (Supplemental Table 1). Interestingly, if the reaction with GSSG was prolonged for ten minutes, only the mixed disulfide Cys94SSG was quantitatively recovered while no other cysteines were found to be linked with GSH. This indicates that the natural Cys94-Cys76 disulfide is the last bridge to be formed in accordance with previous observations^11,14,24,25^, and that all other glutathionylated cysteines rapidly forms disulfides; among them, only a few being correct, as shown by the partial recovery of activity in the absence of GSH (Supplemental Fig. 1).

### The interaction of Lyz_red_ with GSSG displays specificity

The remarkable enhanced reactivity of Cys94 toward GSSG (about 3000 times) appears specific for this natural disulfide. In fact, while the absolute reactivity of a single residue of Lyz_red_ for cystine is similar and even higher compared to the one for GSSG (*k* = 770 and 600 M^−1^ s^−1^, respectively) the enhanced reactivity toward cystine is only about 60 times and null toward cystamine (Fig. 2c). Checking these two disulfides, GSH was chosen as reference free thiol as its p*K*_a_ (9.2) and its structure resembles a protein cysteine. We underline that the different kinetic properties of cystine and cystamine in their interaction with GSH are mainly due to electrostatic factors. A further interesting behavior of Lyz_red_ was observed with organic hydrophobic compounds like DTNB, a well-known thiol reagent. The reaction is very fast at pH 7.4, so we preferred to perform all experiments at pH 5.0 and at low DTNB concentrations. Under these conditions DTNB reacts with an apparent near-monophasic behavior involving most of the protein cysteines. The average second order kinetic constant was 3200 M^−1^ s^−1^ i.e. 160 times higher than the one of GSH at the same pH value (Fig. 2a,c). This reagent gave other important informations. In fact, the reaction with sub-stoichiometric amounts of DTNB (1 mole DTNB with 1 mole of Lyz_red_, containing 8 reduced cysteines) releases 2 moles of TNBS^−^ (Fig. 3b) demonstrating that, unlike Lyz-S-SG, the mixed disulfide Lyz-S-S-TNB rapidly evolves giving the intramolecular disulfide (Fig. 3c).

Hyper-reactivity was also found by reacting Lyz_red_ with 1-chloro-2,4-dinitrobenzene (CDNB) and NBD-Cl, two well-known alkylating agents (Fig. 2a) involving four cysteines of Lyz_red_ with enhanced reactivities spanning from 2200 to 360, respectively (Fig. 2a,c). Electrostatic factors (i.e., at pH 7.4 lysozyme is positively charged, NBD-Cl and CDNB neutral and negatively charged, respectively) are the probable cause of the different increased reactivities of Lyz_red_ toward these two organic reagents.

**Figure 4:**
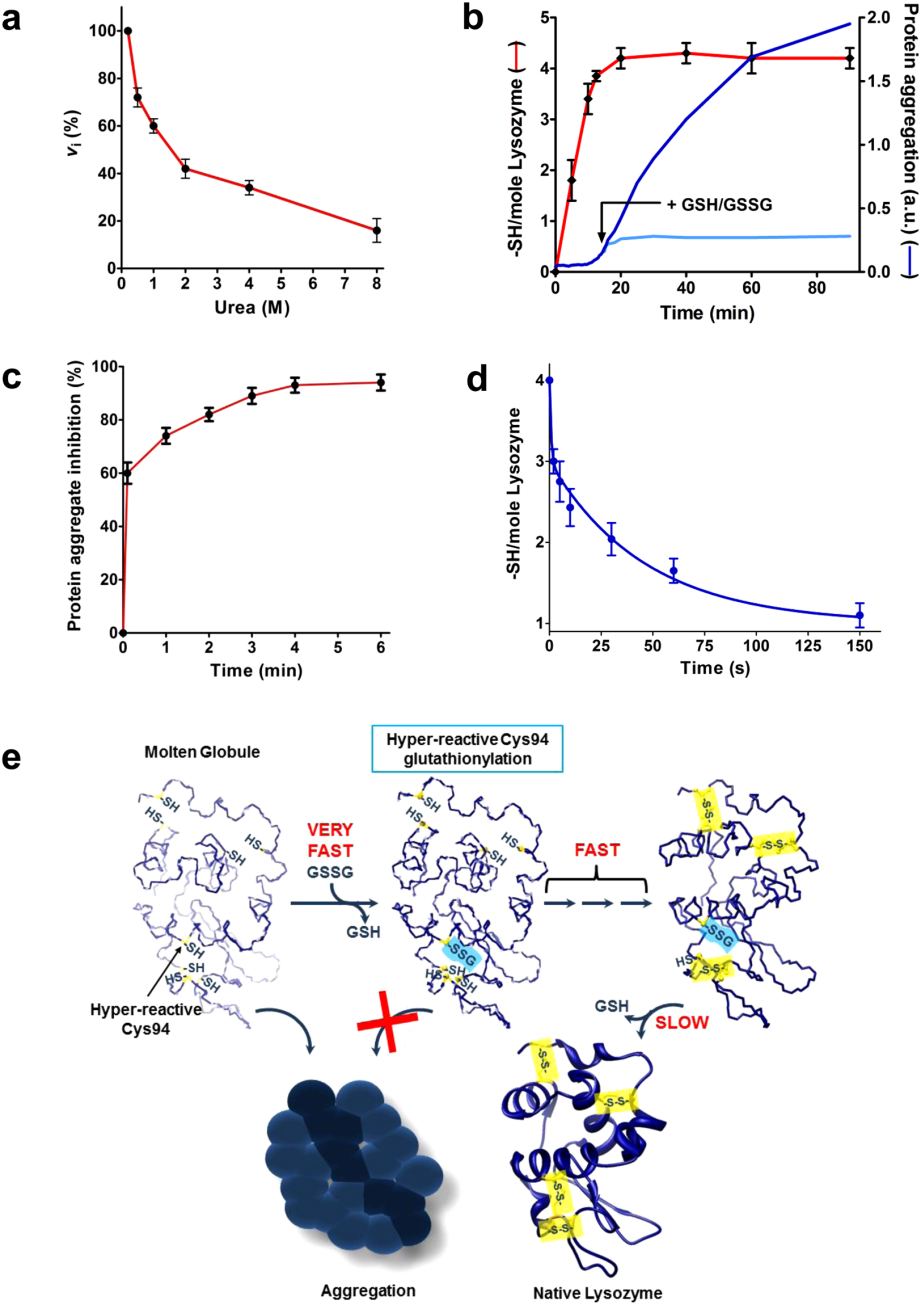
The effect of urea on the hyper-reactivity of cysteines in Lyz_red_ and the inhibition of the aggregation of the hemi-reduced lysozyme by GSH/GSSG. (**a**) Inhibition of the hyper-reactivity of cysteines in Lyz_red_ toward DTNB by variable urea concentrations (1.25 µM Lyz_red_ was reacted with 20 mM DTNB at pH 5.0). (**b**) *Red line*: Hemi-reduction of native Lyz (6 µM) incubated with 60 µM DTT in 0.01 M borate buffer pH 8.5 without urea. No aggregation occurs during the reduction at this pH. *Blue line*: Aggregation of the hemi-reduced Lyz when the pH was lowered to 7.4 with phosphate buffer 0.1 M. *Light blue line*: Inhibition of aggregation by addition of GSH/GSSG (2 mM/0.4 mM). (**c**) Expanded kinetics of inhibition of the protein aggregation reported in **b** after addition of GSH/GSSG (2 mM/0.4 mM). (**d**) Disappearance of three hyper-reactive sulfhydryls in the hemi-reduced Lyz (1.25 µM) after reaction with 0.4 mM GSSG at pH 7.4. Note that the first sulfhydryl reacts within 7 seconds. Errors are reported as S.D. from three independent experiments in panels (**a**–**d**). (**e**) Scheme of the protection mechanism of Lyz_red_ to avoid the protein aggregation. Note that the natural Cys76-Cys94 is the last bridge to be formed^11,14,24,25^.

All these peculiar and never described kinetic properties of cysteines in Lyz_red_ are surprisingly enabled by an only partially folded structure which is visible in the CD spectrum (Supplemental Fig. 2) and possibly identified as the so-called molten-globule conformation. Only about 30% of the native secondary structures are present in this primordial build. However, a residual secondary structure is still present in 8 M urea (Supplemental Fig. 2), in agreement with the presence a residual hyper-reactivity toward DTNB (Fig. 4a).

### Cysteines of partially reduced lysozyme display also hyper-reactivity

Partial reduction of lysozyme, which could occur *in vivo*, for example caused by an excess of antioxidant drugs^26,27^, also triggers protein aggregation. After incubation of the enzyme with low dithiotreitol (DTT) concentration *i.e*. lysozyme:DTT (1:2) without urea, only one disulfide was reduced. The corresponding two sulfhydryls do not display any relevant hyper-reactivity and no protein aggregation occurs within ten min. On the basis of previous studies, the first disulfide that undergoes reduction may be the one involving Cys6 and Cys127^28,29^. Conversely, the subsequent reduction of a second disulfide, obtained using an higher DTT concentration (1:10), discloses one cysteine with strong hyper-reactivity toward DTNB and other reagents (Fig. 2a). The appearance of this residue is also accompanied by a massive protein aggregation (Fig. 4b). As above observed for the fully reduced lysozyme, the addition of GSH/GSSG mixture in a few seconds lowers to about 40% the velocity of the protein aggregation and a complete inhibition is achieved within 3–4 min (Fig. 4b,c). The parallel disappearance of one hyper-reactive sulfhydryl in a few seconds (Fig. 4d) demonstrates that this phenomenon is due to the glutathionylation of a single cysteine, as observed for Lyz_red_. An estimation of the reactivity of the hyper-reactive cysteine toward GSSG displayed similar kinetic properties found for Cys94 in the fully reduced enzyme (Fig. 2a) and thus a reasonable identification of this residue with Cys94. Even in this case, the enhanced reactivity of only a single cysteine toward GSSG is a critical property which opposes the deleterious event of the protein aggregation. The kinetic properties of this and of other interacting cysteines with different reagents are reported in Fig. 2a.

## Conclusions

In conclusion, we have described a completely new property inherent the reduced and hemi-reduced forms of lysozyme which seems to be acquired during evolution to prevent deleterious protein aggregation both in the nascent phase or, once in the native state, after accidental reductive stress (see the representative scheme in Fig. 4e). The extraordinary enhanced reactivity of Cys94 toward GSSG indicates that the glutathionylation of this residue may represent *in vivo* the primordial event which carries out this protection. Although the reactivity toward cystine is similar, GSSG is the prime target *in vivo* due to its higher concentration in the endoplasmic reticulum (0.4 mM), compared to the cystine level (about 0.01 mM). Following endoplasmic reticulum redox perturbations, blocking the free thiol group of Cys94 with GSSG can prevent lysozyme aggregation during the folding process. The glutathionylated Cys94 will then form its native disulfide with Cys76 as a last event during the oxidative folding according to what reported in literature^11,14,24,25^. Interestingly, the analog mixed disulfide Cys95SSG in the human protein has been proposed as a protective group to prevent the formation of an incorrect disulfide bond during the protein folding^30^ and this additional role can be also extended to the corresponding disulfide of the chicken egg lysozyme. Moreover, it is well known that the folded lysozyme released from the cell might undergo occasional reductive stress within the extracellular matrix leading to protein aggregation and disease^7^. Glutathionylation of Cys94 by the GSSG present in the extracellular matrix might slow down the aggregation rate leading either to the reformation of the native molecule or to proteolytic degradation of the partially reduced lysozyme. Both a lowered p*K*_a_ and a transient Lyz_red_-GSSG complex are the main determinants of the enhanced reactivity toward GSSG found for Cys94 while a lower average p*K*_a_ value alone justifies the moderate increased reactivity of the remaining protein cysteines toward GSSG. An additional favorable hydrophobic interaction between Lyz_red_ and other lipophilic reagents like DTNB, NBD-Cl and CDNB is the probable cause of the widespread relevant hyper-reactivity toward these reagents. A particular interest has been developed in the last years to identify hyper-reactive cysteines and an elegant proteomic assay has been published to profile quantitatively such cysteines mainly involved in catalytic mechanisms^31^. Thus, the discovery of these particular kinetic properties of cysteines only devoted to assume a structural role as disulfides in the native protein, as also recently found for Cys75 in human albumin^17^, is a completely new finding that opens interesting perspectives for the knowledge of molecular mechanisms underlying the correct protein folding during transit in the Golgi, as well as the molecular extracellular events responsible for misfolding diseases^5^ with a particular emphasis to lysozyme amyloidosis, rare non-neuropathic forms of hereditary amyloidosis^32–35^.

## Methods

### Materials

Lysozyme (Lyz) from chicken egg white (about 100,000 U/mg), L-cysteine (Cys), L-cystine, L-glutathione (GSH), oxidized glutathione (GSSG), cysteamine, cystamine, 1-chloro-2,4-dinitrobenzene (CDNB), 5,5′-dithiobis(2-nitrobenzoic acid) (DTNB), 4-chloro-7-nitrobenzofurazane (NBD-Cl), dithiotreitol (DTT), bromopyruvic acid, lysozyme activity kit, pepsin A and all other reagents were from SIGMA-Aldrich (St. Louis, Mo, USA).

### Lysozyme reduction

Lysozyme concentration was evaluated on the basis of an extinction coefficient of 37,970 M^−1^ cm^−1^ at 280 nm. Reduction of lysozyme was performed by dissolving the protein (0.05 mM final concentration) in 0.01 M sodium borate buffer, pH 8.5 with or without 8 M urea and incubating it with variable DTT concentrations (lysozyme:DTT, 1:2, 1:10) at 40 °C. The pH was always adjusted to 8.5 with 0.1 M NaOH. At fixed times, the content of reduced cysteines was evaluated on the basis of the residual DTT calculated using DTNB as –SH reagent (ε_M_ TNBS^−^ = 14,100 M^−1^ cm^−1^ at pH 8.0) after filtration of the mixture on an Amicon Ultra filter with a cut-off of 10,000 Da. Alternatively, the reduced cysteines in Lyz were evaluated by reacting the reduced Lyz (1.25 µM) with 20 µM DTNB at pH 5.0 (ε_M_ TNBS^−^ = 11,800 M^−1^ cm^−1^ at pH 5.0) taking advantage by the hyper-reactivity of all 8 protein cysteines with this reagent (Supplemental Fig. 3). Throughout the paper, the term “reduced lysozyme” (Lyz_red_) refers to that obtained after 40 min of reduction with DTT (lysozyme:DTT, 1:10) at 40 °C with 8 M urea. The reduced protein in 8 M urea shows 7.7 ± 0.3 reduced cysteines and very scarce enzyme activity (about 4%). On the other hand the reduction performed without urea with only two equivalents of DTT (Lyz 1.25 µM, 0.01 M sodium borate buffer pH 8.5, 30 min, 37 °C) reduces only one disulfide while two disulfides are reduced using a ten times molar excess in the same condition. This enzyme is defined in the text as “hemi-reduced” Lyz. DTT stock solutions were titrated with DTNB prior to use.

### Inhibition of Lyz_red_ aggregation by GSH/GSSG mixture

Lyz_red_ used to follow the aggregation event was obtained by a reduction step similar to that above reported but using higher Lyz concentration i.e. 0.25 mM in 8 M urea and 2.5 mM DTT in 0.01 M sodium borate buffer, pH 8.5. The reduction progress and the final number of reduced cysteines were similar to those observed using more diluted conditions. Aggregation of Lyz_red_ was observed by incubating the protein (6 µM) with a 0.2 M final concentration of urea in 0.01 M potassium phosphate pH 7.4. The turbidity due to protein aggregation was followed at 600 nm. The effect of GSH/GSSG (2 mM/0.4 mM) was observed by adding these reagents after 5 seconds after the beginning of the aggregation.

### Reactivity of Lyz_red_ cysteines toward GSSG

The interaction of Lyz_red_ with GSSG at the level found in the endoplasmic reticulum was measured by incubating 1.25 µM of Lyz_red_ in 0.2 M urea with 0.4 mM GSSG in 0.1 M potassium phosphate buffer, pH 7.4, 25 °C. At various times the loss of hyper-reactive cysteines due to reaction with GSSG was evaluated using DTNB as –SH titrant. Reactivity of free cysteine with GSSG was evaluated by following the disappearance of cysteine using bromopyrivuc acid^17^.

### Titration of GSH with NBD-Cl and glutathione transferase isoform P1-1 (GSTP1-1)

GSH released after reaction of Lyz_red_ (1.25 µM) with 0.4 mM GSSG at pH 7.4 for 10 seconds, was measured as follows; 0.5 ml of the incubation mixture was acidified to pH 5.0 with a few drops of formic acid to stop any redox reaction. The solution was passed through an Amicon Ultra (10 K Membrane) (Millipore, Cork, IRL). The filtrate was incubated with 0.02 mg GSTP1-1 and 0.2 mM NBD-Cl.

### Alternative titration of GSH coming from the interaction of Lyz_red_ with GSSG

GSH released after reaction of Lyz_red_ (1.25 µM) with 0.4 mM GSSG at pH 7.4 after fixed incubation times (25 °C) was measured as follows: after reduction of 0.1 mM Lyz with 1 mM DTT in 0.01 mM borate buffer, pH 8.5 (50 min at 50 °C) containing 8 M urea, the solution was passed through a Sephadex G-25 column (1 × 20 cm) equilibrated with 20 mM sodium phosphate buffer, pH 7.4 containing urea 2 M and 1 mM EDTA. The collected protein (40 µM) is now without DTT. The protein was then diluted up to 4 µM with 20 mM potassium phosphate buffer, pH 7.4 containing 0.2 M urea and 0.1 mM EDTA. GSSG was then added (0.4 mM final concentration). At fixed times aliquots were acidified to pH 5 with acetate buffer (0.1 M final concentration) and 0.5 ml centrifuged at 15000 × g on an Amicon Ultra (10 K Membrane) (Millipore, Cork, IRL). The filtrate was brought to pH 8 with phosphate buffer and GSH titrated with DTNB.

### p*K*_a_ determination

The average *pK*_a_ of cysteines in Lyz_red_ was calculated on the basis of the different reactivity of these residues (1 µM Lyz_red_) with DTNB (20 µM) in 0.02 M Britton-Robinson buffers at different pH values (from 4.0 to 8.0). Below pH 7.0, appropriate TNBS^−^ extinction coefficients at 412 nm were considered. A similar experimental approach was performed using NBD-Cl (20 µM with 1 µM of Lyz_red_) as thiol reagent at different pH values. The cysteine-NBD adduct absorbs at 419 nm (ε_M_ = 13,000 M^−1^ cm^−1^)^36^. *v*_0_ values are the initial velocity of reaction of Lyz_red_ with DTNB or NBD-Cl as calculated spectrophotometrically in continuous at 412 nm and 419 nm, respectively. Observed initial velocities (*v*_0_), normalized to that calculated at full deprotonation (*V*_max_), were plotted against pH values. p*K*_a_ values were calculated using a curve fitting analysis.

### Circular dichroism and fluorescence analysis

CD spectra of native Lyz, Lyz_red_, and hemireduced Lyz were measured at 1.25 μM concentration in 10 mM potassium phosphate buffer pH 7.4, 25 °C. The setting panel of the spectropolarimeter Jasco J-600 was: slit 2 nm, sensibility 50 mdeg, range 245-200 nm, resolution 0.2 nm; using a quartz cuvette of 0.1-cm light path. The fluorescence measurements were performed in continuous on a Fluoromax-4 Horiba spectrofluorometer with slits 5 nm, excitation wavelength 295 nm, emission wavelength at 340, temperature 25 °C, with a quartz cuvette of 1-cm light path. The apparatus was provided with a rapid mixing device.

### Reactivity of Lyz_red_ toward several disulfides and thiol reagents

The reactivity of the sulfhydryl groups of Lyz_red_ toward DTNB was evaluated spectrophotometrically in continuous at 412 nm where TNBS^−^ absorbs. The experiments were performed both in the presence of residual DTT used to reduce the native Lyz (with minor corrections) or without DTT after G-25 Sephadex column, obtaining coincident results. In fact protein cysteines are 850 times (one cysteine) and 20 times (six cysteines) more reactive than DTT. In a typical experiment 1.25 µM Lyz_red_ was reacted with 20 µM DTNB in acetate buffer pH 5.0. The kinetics follows an near-hyperbolic behavior so second order kinetic constants were evaluated on the basis of initial rate or of t_1/2_ at different DTNB concentrations. The reactivity toward cystine and cystamine was determined by reacting Lyz_red_ (1.25 µM) with 0.4 mM of both disulfides in 0.1 M potassium phosphate buffer pH 7.4. At fixed times aliquots were placed in 0.1 M acetate buffer, pH 5.0 and the disappearance of the hyper-reactive cysteines of Lyz_red_ was determined using DTNB as titrant. The reactivity toward CDNB was evaluated spectrophotometrically in continuous at 340 nm where the Cys-DNB adduct absorbs (ε_M_ = 9,600 M^−1^ cm^−1^)^17^. In a typical experiment Lyz_red_ (1.25 μM) was reacted with variable CDNB concentrations (from 0.5 mM to 2 mM) in 0.1 M potassium phosphate buffer, pH 7.4. A slight turbidity due to the CDNB modified enzyme was subtracted by each determination. The reactivity of Lyz_red_ toward NBD-Cl was determined as above described for CDNB using NBD-Cl (from 10 µM to 60 µM). The reaction was followed spectrophotometrically at 419 nm were the Cys-NBD adduct absorbs (ε_M_ = 13,000 M^−1^ cm^−1^)^36^. Second order kinetic constant for the reaction of free Cys toward GSSG (0.7 M^−1^ s^−1^) was determined previously^17^ (see Fig. 2a). The theoretical kinetic constant for an unperturbed protein cysteine was calculated by considering the p*K*_a_ value of its sulfhydryl group = 9.1 instead of 8.53 of the free amino acid^23,37^. Second order kinetic constants for the reaction of free GSH toward all other reagents were calculated on the basis of initial velocity of the reaction of 10 µM of GSH (1 mM with CDNB) with each reagent in the same condition used for the assay with Lyz_red_. The velocity of the reaction of GSH with cystamine and cystine was evaluated by determining at fixed times the amount of cysteine or cysteamine released as a consequence of the reaction. Cysteamine and cysteine were determined on aliquots after reaction with 1 mM bromopyruvate. The reaction is almost instantaneous and the observed product is a cyclic sulfur compound (lanthionine ketimine and aminoethylcysteine ketimine) absorbing at 296 nm (ε_M_ = 6,200 M^−1^ cm^−1^)^38^.

### Effect of urea concentration on the hyper-reactivity

The effect of urea on hyper-reactivity of Lyz_red_ was assayed using DTNB as thiol reagent. In a typical experiment, Lyz_red_ (1.25 µM) was incubated with 0.01 M potassium phosphate buffer, pH 7.4, in the presence of variable concentrations of urea (from 0.2 M to 8 M). After five min incubation the rate of reaction with DTNB (20 µM) was measured spectrophotometrically at 412 nm in 0.1 M acetate buffer pH 5.0 (25 °C).

### Aggregation of the hemi-reduced Lyz

Concentrated hemi-reduced Lyz (6 µM) was produced by incubating Lyz with ten molar excess of DTT at pH 8.5. At the end of the reduction only two disulfides are reduced and a relevant aggregation occurs at pH 7.4 (see Fig. 4b).

### Lysozyme activity

Activity of lysozyme was assayed by the lysozyme detection kit (Sigma-Aldrich, St. Louis, MO, USA) which uses *Micrococcus lysodeikticus* cell suspension as substrate^39^.

### Mass spectrometry identification of hyper-reactive cysteine

Lyz_red_ (1.25 μM) was incubated with GSSG (0.4 mM) in 0.01 M potassium phosphate buffer, pH 7.4. The reaction was stopped after 10 seconds or 10 min by adding 0.25 mM bromopyruvate which alkylated residual protein cysteines within 1–2 sec. Then the samples were lyophilized. A Lyz_red_ solution (1.25 μM) was immediately alkylated with bromopyruvate and used as control. Samples were resuspended in 0.2% TFA and desalted by reversed-phase HPLC on a Phenomenex Jupiter C4 column (250 mm × 2.0 mm, 300 Å pore size) with a linear gradient from 10% to 95% of solvent B (0.07% TFA in 95% acetonitrile) in 30 min, at a flow rate of 200 μL/min using an Agilent Technologies 1100 HPLC (Agilent Technologies, USA). Protein fractions were collected and lyophilized. Controlled pepsin hydrolysis was carried out by dissolving the samples in 5% formic acid, pH 2.5 and adding pepsin at an enzyme to substrate ratio of 1:300 w/w at 37 °C for 2 hours. Sample was then lyophilized, resuspended in 0.2% formic acid and directly analyzed by nanoLC/MS-MS on an LTQ-XL Orbitrap mass spectrometer equipped with a nanoHPLC (ThermoFisher, USA). Peptides containing modified cysteine residues were selected using the ion extraction chromatograms of the corresponding doubly and triply charged ions and the assignments were confirmed by manual inspection of their fragmentation spectra.

### Statistical and graphical analysis

Data are represented as means ± standard deviation (S.D.). Statistical analysis was performed using computer software packaged MedCalc (Mariakerke, Belgium). The graphic and results visualization were obtained using GraphPad Prism software (La Jolla, CA, USA).

## Electronic supplementary material

Supplementary Information
