# Supplementary material for: The extreme hyper-reactivity of Cys94 in lysozyme avoids its amorphous aggregation
Source: Sci Rep. 2018 Oct 30;8:16050. doi: 10.1038/s41598-018-34439-y (PMC6207692; doi:10.1038/s41598-018-34439-y)
Supplement: Supplementary file 1 — Supplementary Information [file 41598_2018_34439_MOESM1_ESM.pdf]

Supplementary Information

The extreme hyper-reactivity of Cys94 in lysozyme avoids its amorphous aggregation

Alessio Bocedi[1], Giada Cattani[1], Claudia Martelli[2], Flora Cozzolino[3], Massimo Castagnola[2], Pietro Pucci[3] and Giorgio Ricci[1]

[1]Department of Chemical Sciences and Technologies, University of Rome “Tor Vergata”, Rome, Italy. [2]Istituto di Biochimica e Biochimica Clinica, Università Cattolica and Istituto di Chimica del Riconoscimento Molecolare, Consiglio Nazionale delle Ricerche, Rome, Italy. [3]CEINGE Biotecnologie Avanzate and Department of Chemical Science, University of Naples “Federico II”, Naples, Italy.
Correspondence and requests for materials should be addressed to G.R. (email: riccig@uniroma2.it)

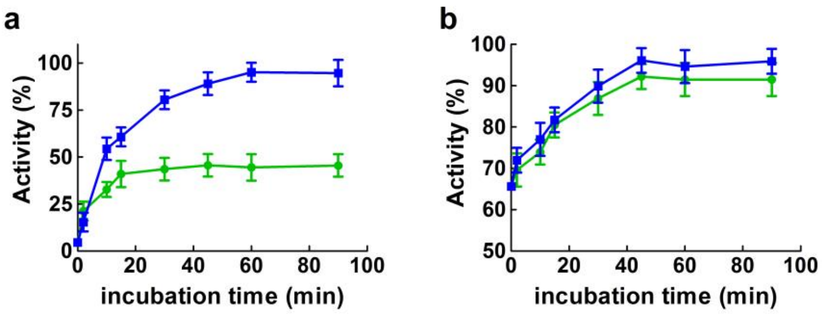

Supplemental Figure 1. (**a**) Activity restoration of Lyz_{red} by GSH/GSSG (2 mM/0.4 mM) (37 °C) (*blue line*). Activity restoration of Lyz_{red} by only 0.4 mM GSSG pH 7.4, 37 °C (*green line*). (**b**) Activity restoration of hemi-reduced Lyz incubated at pH 7.4 by GSH/GSSG (2 mM/0.4 mM) (37 °C) (*blue line*) or by only 0.4 mM GSSG (*green line*). Errors are reported as S.D. from three independent experiments.

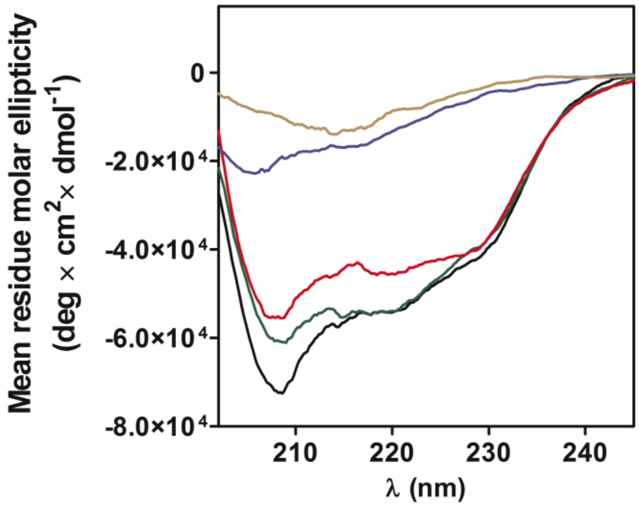

Supplemental Figure 2. CD-spectra of native lysozyme (*black line*), lysozyme with only one reduced disulfide (*green line*), hemi-reduced lysozyme (*red line*), fully reduced Lyz in 0.2 M urea (*blue line*) and fully reduced Lyz in 8 M urea (*yellow line*). The peculiar disappearance of ordered structure in the lysozyme lacking a single disulfide (centered at 208 nm) and that observed in the hemi-reduced lysozyme (centered in the 212-223 nm region) suggests that a random partial reduction of the four disulfides did not occur but that two different disulfides are reduced sequentially.

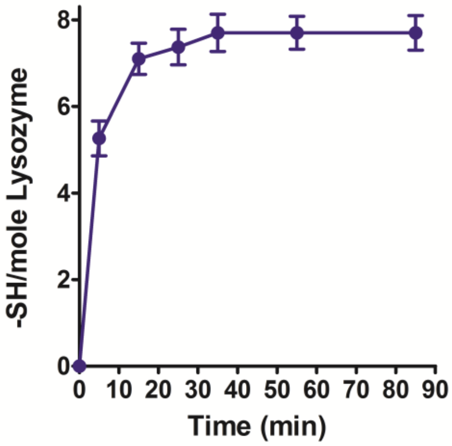

Supplemental Figure 3. Kinetics of Lyz reduction with ten molar excess of DTT. 1.25 µM Lyz was reacted with 12.5 µM of DTT in 0.01 M borate buffer pH 8.5 at 40 °C. The pH was adjusted at 8.5 with 0.1 M NaOH. At various times the reduced protein cysteines were determined by centrifuging aliquots on Amicon Ultra (10K Membrane) (Millipore, Cork, IRL) and titrating the filtrated DTT with DTNB at 412 nm. Same results were obtained by reacting 25 µL of the solution with 20 µM DTNB at pH 5.0. All hyper-reactive cysteines of Lyz_{red} reacted with DTNB in less than 2 min. The final absorbance was subtracted by the very small contribution of the residual excess of DTT. After 40 min the maximum of the reduction (7.7 ± 0.3 -SH/mole Lyz_{red}). Errors are reported as S.D. from ten independent experiments.

Supplemental Discussion

Effect of a lowered pK_a of the sulfhydryl group of a cysteine on the rate of its reaction with disulfides.

The only active form of a thiol group in its reaction with disulfides is its deprotonated form:

$$RSH + R'SSR' \rightarrow \text{no reaction}$$

$$RS^- + R'S\text{-}SR' \leftrightarrow RS\text{-}SR' + RS^-$$

An unperturbed protein cysteine has a pK_a = 9.1 (Ref. 23). Thus

$$\frac{[\mathrm{CysS^-}][\mathrm{H^+}]}{[\mathrm{CysSH}]} = 10^{-9.1}$$

The fraction of dissociated sulfhydryl is:

$$\frac{[\mathrm{CysS^-}]}{[\mathrm{CysS^-}] + [\mathrm{CysSH}]} = \alpha = \frac{\frac{[\mathrm{CysS^-}]}{[\mathrm{CysSH}]}}{\frac{[\mathrm{CysS^-}]}{[\mathrm{CysSH}]} + 1} = \frac{10^{\mathrm{pH}-\mathrm{p}K\mathrm{a}}}{1 + 10^{\mathrm{pH}-\mathrm{p}K\mathrm{a}}}$$

At pH 7.4, $\alpha = 2.0 \times 10^{-2} = 0.020$

Thus a lowered pK_a of the sulfhydryl group which makes almost fully dissociated a protein cysteine (α = 1) at physiological pH 7.4 (for example K_a < 6.0) cannot cause a kinetic increase higher than 50 times. This represents the maximum kinetic incremental factor expected for an “acidic” cysteine in its reaction with disulfides.

Supplemental Table 1

LC-MS/MS analysis of controlled pepsin hydrolysis of Lyz_{red} treated with 0.4 mM GSSG at pH 7.4 for 10 s and then alkylated with 0.25 mM bromopyruvic acid

Cys residue	Peptides	MH^+	Ions detected	Assignment[a]
6	1-8	1037.52	519.26^{++}	Cys6-Pyr
30	29-38	1201.51	601.25^{++}<br>1201.51^{+}	Cys30-Pyr
64	57-75	2346.12	782.71^{+++}	Cys64-Pyr
64, 76, 80	57-83	3319.45	1107.15^{+++}	Cys64-Pyr<br>Cys76-Pyr<br>Cys80-Pyr
64, 76, 80	63-83	2535.04	1268.02^{++}	Cys64-Pyr<br>Cys76-Pyr<br>Cys80-Pyr
**94**	**84-108**	**2886.27**	**1443.64^{++}**<br>**962.76^{+++}**	**Cys94-SSG**
115	108-122	1875.88	625.96^{+++}	Cys115-Pyr
127	123-129	989.49	495.25^{++}	Cys127-Pyr

[a] Cys-Pyr: alkylation with bromopyruvic acid; Cys-SSG: mixed disulfide with glutathione
